# A Practical Treatise on Mechanical Dentistry

**Published:** 1861-01

**Authors:** 


*A Practical Treatise on Mechanical Dentistry.* By JOSEPH RICHARDSON, D. D. S., M. D.; Professor of Mechanical Dentistry in the Ohio College of Dental Surgery. In one octavo volume, pp. 427, with one hundred and ten illustrations. Philadelphia: Lindsay & Blakiston, 1860.

We believe this to be the *first* separate extended treatise on this particular department of dentistry that has ever graced American authorship. It is with pleasure that we have cursorily glanced over its pages and subject-matter, and are enabled to speak in general terms commendatory of the work.

We esteem it a privilege here in being able to express our gratification for the manifest enthusiasm that impels to the production of such a work—a comprehensive one having been long in demand. Emanating from the very authentic source that it does, it could not be well other than what it is—a valuable adjunct to dental literature.

The work before us for review is arranged in two parts. *Part First* includes a description of the modes of applying *heat*, the different metals brought into requisition for dental laboratory purposes, and their metallurgic treatment.

*Part Second* is devoted to *artificial dentures*, and includes, in the order of arrangement, the general preparatory treatment of the mouth; the whole range of laboratory operations legitimately pertaining to the construction of artificial dentures; the different bases employed for dental substitutes; the manufacture of block-teeth, materials used in their preparations, together with defects of the palatial organs and their *artificial* remedial means.

In the first chapter he speaks of blow-pipes, their construction and use; of lamps, furnaces and fuel for baking teeth, etc.

The second chapter is upon gold, the sources from which it is obtained, its geological situation, its properties, how influenced by alloying, and properties of the alloys of gold.

Third chapter is devoted to the refining of gold. Of "sweepings" he says, "This form embraces many impurities, earthy and metallic, and should first be thoroughly washed to remove the earthy constituents," etc. I may remark that, inasmuch as in the washing of such sweepings, a loss of metal would undoubtedly result, a better method would be to fuse together the sweepings and substances hereinafter mentioned, in the following proportions: Of sweepings, eight parts; chloride of sodium, four parts; impure carbonate of potassa, four parts; impure super tartrate of potassa, one part; and nitrate potassa, half part. Mix them thoroughly together, and melt in a crucible. The crucible with its contents should remain in the fire for some time, in order to secure a complete separation of the metals from the extraneous matter.

In chapter fourth, when speaking of the quality or fineness of gold plate to be introduced into the mouth as a base for dental substitutes, he says: "But, it must be remembered that, in addition to corrosive agents introduced into the mouth from without, a variety of diseases, local and constitutional, effect important changes in the otherwise bland and innoxious fluids contained therein, which, from being alkaline or neutral, become more or less acidulated. Indigestion, with acid eructations, gastro-enteritis, ague, inflammatory and typhoid fevers, brain affections, eruptive diseases, rheumatism, gout, etc., are some of the local and constitutional disorders almost uniformly imparting to the mucous and salivary secretions an acid reaction. These readily attack the impoverished gold too frequently employed as a base for artificial teeth; and, as a natural sequence to such practice, we find supervening inflammation of the mucous membrane and gums, apthous ulcers, gastric irritation, general nervous disorders, decay of the teeth, fœtid breath, disagreeable metallic taste in the mouth," etc.

Fifth chapter, when speaking of the required forms of metal for dental purposes, a *fact* is set forth worthy of note to the metallurgist at all times. He says: "It not unfrequently happens that, at the first pouring, the metals arrange themselves in the ingot in accordance with the density of the several components; those of the greatest specific gravity passing to the bottom, and the lighter metals remaining above."

The phenomenon here presented seems most conclusive to our mind to be *more* the result of *special affinity* of the components, than of mere specific gravity. By reason of the above (whether the results of specific gravity or of special affinity), in the *assay* of the precious metals, where like combinations are required, granulation is resorted to in order to avoid this objectionable condition which sometimes ensues in the congelation of the ingot metal.

In this chapter is contained various formulas for the different standards of plate and of solders, rules for alloying gold, raising as well as reducing the fineness; also, a comprehensive table, indicating the weight, fineness and value of the coins of different nations.

The sixth chapter treats of silver; first, of its general proportions, then its alloys, next its reduction to the required forms, and lastly formulas for silver solders.

He speaks of the employment of silver in the mouth as a base for artificial teeth, as "having been regarded by many as unsafe and injudicious," and furthermore: "If used at all, therefore, it should be reduced with the least practical amount of copper; or, what is better, pure silver should be reduced with platinum alone, in sufficient quantities to impart to the plate an adequate degree of strength and elasticity. From three to five grains may be added to one pennyweight of pure silver. This is, doubtless, a valuable combination, and is certainly worthy the regard of every dental practitioner.

I notice that under the above head, the author has failed to give the process of refining silver, so as to obtain it in a perfectly *pure* state.

Seventh chapter is given to platinum and the platinoid metals. First, the general properties of platinum, its requisite condition for dental uses ; its welding, forging and general working ; its alloys ; and, lastly, the platinoid metals, as palladiums, iridiums, etc.

Eighth chapter is upon aluminium. We find here that, while its general properties, alloys, etc., are spoken of in full, no well founded hope is given that it will ever be of practical utility in dental practice.

The ninth chapter is given to a consideration of some of the base metals, as copper, zinc, lead, tin, antimony and bismuth and their alloys; affording such general knowledge as will be found indispensable in the subsequent laboratory work of *die-making.*

Chapter tenth, in some general remarks upon alloys and alloying, he says : "In alloying three or more metals differing greatly in fusibility, or that have but little affinity for each other, it is better to first unite those which most readily combine and afterward then with the remaining metal or metals."

In addition to the above we would furthermore suggest, as a practical rule in *all* combinations of metal, union by pairs, having, at the same time, also proper regard to their affinity.

*Part Second,* chapter first, deprecating the practice of inserting an artificial denture over diseased roots of teeth, where the gums are already in an inflamed condition, he remarks : "Soon becomes (*i. e.*, the denture,) a source not only of annoyance and discomfort to the patient, but is rendered, in a degree, insufficient in the performance of some of its most important offices. There is, besides, a perpetual and cumulative aggravation of the morbid conditions, and sooner or later irretrievable destruction of the remaining natural organs will be induced. These consequences can not be wholly averted by the most skillful manipulation, but they may be greatly magnified by a defective execution of the work, or by a faulty adaptation of the appliance to the parts in the mouth."

He then, in plain terms, indicates the duty of the practitioner in relation to the above practice, and the obligations resting upon him for the faithful discharge of such duty, and says: "He should be careful to guard himself against the imputation of incompetency or bad faith, by being peremptory and unyielding in his demands upon the patient to submit to the necessities and just requirements of the case," etc.

We are pleased to see the author come boldly up to this *true* stand-point, assuming the position that every truly worthy practitioner should, commanding the respect and honor to which his profession but justly entitles him.

Of the surgical treatment of the mouth, subsequent to the removal of teeth, he says: "Immediately after the extraction of the teeth, therefore, any flaps of gum hanging loosely around the sockets, should be clipped off, and the sharp, protruding portions of the processes cut away with excising forceps," etc.

From this we must dissent, preferring rather to rely upon nature to accomplish her work of decomposition and assimilation with a *simple* wound, rather than to render it a *complex* one by "clipping off the flaps of gum," (of which there should be none), and breaking down the "sharp and protruding portions of the processes." Should there be fragmentary portions of the processes visible, then of course, in any case, their removal is clearly indicated.

Where proper regard is had to the removal of teeth and roots of teeth—avoiding, by every possible means, the undue laceration of the soft parts, or fracturing the surroundings—there does not seem to us a necessity for inflicting so severe a *penalty* in order to avoid subsequent inconvenience, or even slight perodical suffering. The *infliction* of pain upon patients should ever be sedulously guarded against.

He says the objection has been urged against the practice of inserting temporary teeth, "that they tend to produce unequal absorption of the parts on which they rest." This view he regards as "mainly speculative," for, as the "ridge recedes in the process of absorption, it is more and more relieved from direct contact with the plate resting against the roof of the mouth, preventing it from following the retreating gums." Observation has led us to believe that, in some cases, the *reverse* is true; being cognizant of cases showing a striking conformation of the alveolar ridge, both superior and inferior, even at the late period of three years after inserting the temporary teeth. This condition in the upper jaw seems to us the result partly of change in the tissue from pressure of the plate against the roof of the mouth, and partly from the yielding of plate favoring *its* adaptation to the ridge. This last, however, will not apply to the inferior ridge, where we have frequently seen the same result. We do not wish, however, to be understood as not advocating the practice of inserting these substitutes. On the contrary, we deem their general utility and approximation to subserve the natural wants too apparent to be ignored.

Chapter second—preparatory to a description of taking impressions of the mouth, he very appropriately and truthfully favors the importance of this preliminary step toward the construction of the dental substitute. We would direct attention to another preliminary which is omitted, and that is a careful and thorough examination of the mouth. We conceive to be of the first importance that the operator should note particularly all parts to be included in the impression, such as form and position of teeth (when remaining), the texture of the alveolar ridge, arch, etc.

After speaking of the substances for taking impressions, he goes on with a minute and comprehensive detail of taking the various impressions of the mouth. We notice in this chapter, under "taking impressions with plaster," omission of some *little things;* but, nevertheless, such as we think contribute much toward perfection in the manipulation we are considering. First is, after introducing the cup (containing the plaster) into the mouth, distending the cheeks with the tongue-holder, conjointly with "extending and drawing outwardly the lip." Then, when about to take an under impression, direct the patient, when the cup has been introduced, to elevate the tongue toward the roof of the mouth, allowing it to assume its proper position as the cup is pressed down upon the alveolar ridge; and, furthermore, as in the case of taking an upper impression, alternately distend the cheeks with tongue-holder. In this way the loose integuments and muscular folds will be excluded from the impression, which otherwise so frequently cause defective impressions.

Chapter third—in obtaining plaster models from impressions in wax, he advises that the surface of the impression should be thinly coated with oil; and, furthermore, speaks of rendering the model imperfect by the *injudicious* use of the oil. For the self-same reason that he urges to its *improper* use, we would bring against its use in any case; our preference being most decidedly to "oiling" with *water*—allowing a stream of water to flow into the impression for a few minutes, then, with a sudden throw of the hand holding the impression, the excess of water is displaced, when proceed to fill as described.

We would here suggest, as a necessity for careful examination of the mouth, to which allusion has already been made, that, in case attachments of the frenum linguæ approaches near the prominence of the ridge, the cast at this point should be slightly elevated with wax, so as to provide relief from the pressure of the plate. Or where the position of the anterior mental foramen through which comes the inferior maxillary artery, are such as to admit of pressure by the plate, like provision should be made with wax upon the plaster casts. This is also applicable sometimes to the palatine artery in its passage through the foramen incisivum.

When the model is to be obtained from impression in plaster, he advocates the use of spirit varnish upon the surface of the impression, thereby coating it, then oiling the surface as in the case of wax impression. From this we must beg to differ, believing that more desirable means may be employed; *i. e.*, soaking the plaster impression for some fifteen or twenty minutes in water, or what is more preferable, soap and water. After there is a complete saturation of the plaster with the liquid, it should *at once* be surrounded with the wax-cloth, and filled as described.

Chapter fourth is devoted to metallic dies and counter-dies, embracing the manner of molding in sand and obtaining the die, then making the counter-die; speaks at length of the essential properties of a die.

Chapter fifth takes up pivoting teeth; describes the various, most recent and improved methods for performing this operation.

Chapter sixth. This opens with remarks upon the use of clasps, which are thorough and practical; then refers to the classes of teeth proper to clasp to, as well as those that should not be clasped; the different form of clasps; variety in form of plates for partial denture retained by clasps; swaging of such plates and attaching clasps thereto.

Chapter seventh. In this chapter he gives a method of ingeniously substituting cylinders of wood and tubed plates for clasps attached to plates. This method, although originating some years since, by Dr. W. M. Hunter, seems not to have gained general approval.

Chapter eighth. In this is given the combined use of metallic plate, pivot and clasp for special cases; had been of great utility indeed when properly applied.

Chapter ninth. Plates retained in the mouth by atmospheric pressure. We observe that under this head he does not say anything of the relative size or depth of *cavity*. These are practical points, and therefore it is that we make allusion to them.

For the greatest amount of atmospheric pressure we must needs have the largest possible *surface.* In such plates (the use of which is impl'ed), however, the size of the air-vacuum will depend upon the particular configuration of the mouth. The depth of the cavity, like the size, can be but relative. Where the alveolar arch and ridge are uniformly firm in their texture, the cavity should not be deeper than would be formed by elevating the cast twice the thickness of common card paper. Where the roof of the mouth is comparatively spongy, it should be of greater depth, proportionate in a measure with the yielding tissue.

Another (although in some respects similar) method of forming the chamber, the credit of which I believe is due to Prof. T. L. Buckingham, consists in employing a piece of copper, of the desired shape and thickness, to give form and depth to the cavity, instead of the usual elevation upon the plaster model and metallic die. When the plate, having an orifice corresponding with this piece of copper, has been nearly swaged, a *thin* cap is struck up over the plate and copper. This cap is now trimmed, leaving a narrow margin overlapping upon the plate. After the swaging has been completed, borax and solder should be placed around the proposed line of union of cap and plate, and, with a small iron or steel clamp holding them together, they should be carefully bedded in some dry sand, and the soldering accomplished.

Chapter tenth. Contained in this chapter we have, making antagonizing models of partial dentures; selecting, arranging and antagonizing the teeth; investing, staying, soldering and finishing cases.

For investing cases preparatory to staying and soldering, he recommends a mixture of "plaster, sand and asbestos—two parts of the former and one part each of the latter." From our own knowledge and observation in this particular, we are led to conclude that the proportion of plaster here recommended is too great, believing that so large proportion is calculated to induce warping of plates.

Of finishing or polishing plates, he copies at length an article which he attributes to the pen of Prof. T. L. Buckingham, but which was written by Prof. J. L. Suesserott and published in a dental journal. We may state, in this connection, though somewhat irrelevant, that this peculiar and commendable plan of finishing plate-work, originated, as we are informed, some eight years since with Prof. W. Calvert, which it would have been well for the writer to have noted.

Chapter eleventh. The subject matter of this chapter includes the making of entire upper and under plates; their adaptation to the mouth; securing the proper occlusion of the jaws, etc.; together with rimming plates, making and adjusting spiral springs, and final adjustment of the complete denture in the mouth.

Chapter twelfth. The author here enters upon another and materially different branch—different as relates both to material employed and manipulation.

First, we find the component parts of tooth-porcelain considered. In speaking of these substances, we notice he fails to give practical indications for their selection, and for ascertaining their absolute quality by suitable tests. The most important one of these, and the one entering most largely in the composition of porcelain, is feld spar. It varies very materially in quality, even that obtained from the same locality, and yet, to the unpracticed, the difference in its general aspect would not be distinguishable. It is a double silicate of alumini and *alkali.* The alkali may be soda or potassa, and upon either will depend the quality of the spar, good or bad. He says the spar "is prepared for use in the same manner as silex." So it is; but it should never be ground near so fine—not finer usually than will readily pass through a number nine bolting-cloth seive.

Chapter thirteenth. In this we find described the manner of combining materials for body and enamels; making models and moulds; the process of carving, enameling, baking and mounting *block-teeth.*

There are other methods of making block-teeth not mentioned here. One somewhat peculiar in itself, and possessing no inconsiderable merit, is that invented by Prof. W. Calvert, and consists in enameling and moulding the blocks. The complexity of the process, together with want of time and space, forbids that we should further extend our remarks. We find him omitting the manufacture of single teeth altogether.

Chapter fourteenth. In this is contained a full descriptive treatise on the manufacture and use of what is termed *continuous gum work.*

In this style of work, platinum is employed as the base-plate, formed and adapted to the mouth as plates usually are, after which the silicious material embodying the teeth, is baked or semifused upon the plate.

Chapter fifteenth. In this is described at length the construction of dental substitutes where, as a base, the "vulcanite" material is brought into one; the vulcanizing apparatus; directions for vulcanizing, finishing, etc.

Chapter sixteenth. He here considers a method of constructing cases of artificial teeth, termed "cheoplasty." This method, although of recent origin, has not received the approval of the profession generally.

The concluding chapter is mostly a quotation from the pen of C. W. Stearns, surgeon, formerly of London, who treats ably upon the "defects of palatine organs, and their treatment by artificial means."

The general style of this work and the substantial manner in which it is gotten up, is especially creditable to its publishers; furthermore, we may say, what has already been said, that in this treatise we have another added to the list of important *text-books.* * * *.

